# Stochastic Episodes of Latent Cytomegalovirus Transcription Drive CD8 T-Cell “Memory Inflation” and Avoid Immune Evasion

**DOI:** 10.3389/fimmu.2021.668885

**Published:** 2021-04-22

**Authors:** Marion Griessl, Angelique Renzaho, Kirsten Freitag, Christof K. Seckert, Matthias J. Reddehase, Niels A. W. Lemmermann

**Affiliations:** Institute for Virology, Research Center for Immunotherapy (FZI) at the University Medical Center of the Johannes Gutenberg-University of Mainz, Mainz, Germany

**Keywords:** antigen presentation, effector memory CD8^+^ T cells, gene expression, immune evasion, latency, latent infection, memory inflation, virus reactivation

## Abstract

Acute infection with murine cytomegalovirus (mCMV) is controlled by CD8^+^ T cells and develops into a state of latent infection, referred to as latency, which is defined by lifelong maintenance of viral genomes but absence of infectious virus in latently infected cell types. Latency is associated with an increase in numbers of viral epitope-specific CD8^+^ T cells over time, a phenomenon known as “memory inflation” (MI). The “inflationary” subset of CD8^+^ T cells has been phenotyped as KLRG1^+^CD62L^-^ effector-memory T cells (iTEM). It is agreed upon that proliferation of iTEM requires repeated episodes of antigen presentation, which implies that antigen-encoding viral genes must be transcribed during latency. Evidence for this has been provided previously for the genes encoding the MI-driving antigenic peptides IE1-YPHFMPTNL and m164-AGPPRYSRI of mCMV in the *H-2^d^* haplotype. There exist two competing hypotheses for explaining MI-driving viral transcription. The “reactivation hypothesis” proposes frequent events of productive virus reactivation from latency. Reactivation involves a coordinated gene expression cascade from immediate-early (IE) to early (E) and late phase (L) transcripts, eventually leading to assembly and release of infectious virus. In contrast, the “stochastic transcription hypothesis” proposes that viral genes become transiently de-silenced in latent viral genomes in a stochastic fashion, not following the canonical IE-E-L temporal cascade of reactivation. The reactivation hypothesis, however, is incompatible with the finding that productive virus reactivation is exceedingly rare in immunocompetent mice and observed only under conditions of compromised immunity. In addition, the reactivation hypothesis fails to explain why immune evasion genes, which are regularly expressed during reactivation in the same cells in which epitope-encoding genes are expressed, do not prevent antigen presentation and thus MI. Here we show that IE, E, and L genes are transcribed during latency, though stochastically, not following the IE-E-L temporal cascade. Importantly, transcripts that encode MI-driving antigenic peptides rarely coincide with those that encode immune evasion proteins. As immune evasion can operate only in *cis*, that is, in a cell that simultaneously expresses antigenic peptides, the stochastic transcription hypothesis explains why immune evasion is not operative in latently infected cells and, therefore, does not interfere with MI.

## Introduction

Mouse models of experimental high-dose systemic cytomegalovirus (CMV) infection, using murine cytomegalovirus (mCMV) to account for host-species specificity of CMVs (reviewed in (1)), have revealed an unconventional kinetics of the immune response. After clearance of productive infection, transient contraction of the viral-epitope specific pool of CD8^+^ T cells is followed by pool expansion selectively for certain viral epitopes during non-productive, latent infection (2–6). This phenomenon is known as “memory inflation” (MI) (for reviews, see (7–11)). MI has prompted the promising idea to use CMVs as vaccine vectors by replacing endogenous MI-driving epitopes with epitopes of unrelated pathogens or tumors to generate enduring and self-enhancing immunological memory [(12–16), reviewed in (17–19)].

The expanding CD8^+^ T-cell population is characterized in the mouse model by the cell surface marker phenotype KLRG1^+^CD62L^-^ and was originally classified as short-lived effector cells (SLEC) (20). A recent study has shown an extended life span of these cells, based on IL15-mediated increased expression of the anti-apoptotic protein Bcl-2, which makes them memory cell-like (21). We have therefore suggested naming these cells inflationary T effector-memory cells (iTEM) to distinguish them from KLRG1^-^CD62L^-^ conventional T effector-memory cells (cTEM) (22).

Although MI is not consistently observed in human studies on the development and maintenance of the memory CD8^+^ T-cell response to natural infections with human cytomegalovirus (hCMV), large T-cell responses can be elicited that remain high or even increase over time, and display a phenotype characterized by an advanced differentiation stage (for recent reviews, see (23, 24)). A difference between experimental models and human infection may relate to latent viral genome load, which is determined by the extent and duration of virus replication and spread, based on the history of primary infection in terms of age at the time of infection, route of infection, initial virus dose, and immune status (25–27). As discussed and proposed by Adler and Reddehase (26), congenital infection that is characterized by an extended period of persistent virus replication and shedding due to an immature immune system, is to be expected to generate a high load of latent viral genomes favoring MI. Clinical investigations to test this hypothesis are pending, not least because of ethical concerns.

Local infection, which is rapidly controlled by the immune response (28), failed to support MI of iTEM in immunocompetent mice (22, 29). In contrast, despite the same virus dose and site of infection, transient immunodeficiency in a model of hematopoietic cell transplantation (HCT) (reviewed in (30)) led to systemic acute infection and eventually to a high latent viral genome load supporting MI of iTEM after CD8^+^ T-cell reconstitution (2, 3, 22, 31). While these parameters can be experimentally preset to support MI in animal models, they are given and mostly unknown variables in humans who have individual histories of natural hCMV infections.

Although CD8^+^ T-cell priming determines the magnitude of MI by generating the epitope-specific cells that can later be re-stimulated (32), MI is programmed by viral latency. Maintained expression of the lead marker of iTEM, namely KLRG1, requires persistent or at least repetitive antigen stimulation. KLRG1 is known to be expressed by CD8^+^ T cells during chronic infections but lost in resolved infections (33). While the immunology of MI is well-characterized (7–11), the source of the viral antigens that drive MI is still under debate. CMV infections are not chronic infections with persistent, though low-level, virus production continuously providing antigen for CD8^+^ T-cell re-stimulation, but become latent as defined by maintenance of the viral genome in certain cell types in absence of virus production [(34), reviewed in (35)]. This definition of latency by Roizman and Sears (36) applies to all members of the herpesvirus family. However, CMVs, like all other herpesviruses, can reactivate from latency to productive, recurrent infection. It has been proposed that frequent reactivation events drive MI by re-expression of antigens that then re-stimulate cells to generate a growing pool of iTEM (37).

This “reactivation hypothesis” of MI is, however, not compatible with reports that showed absence of infectious virus in tissues of latently infected mice, including the lungs that represent the major organ site of CMV pathogenesis, latent viral genome load, and reactivation in the model of neonatal infection and in the HCT model (25, 38, 39). Importantly, absence of infectious virus was confirmed by ultrasensitive detection methods that by far exceeded the sensitivity achieved by methods used for routine quantitation of infectious virus (34). Productive reactivation was in fact never observed to occur spontaneously in latently infected, immunocompetent mice, but only after experimental depletion of immune cell subsets or after general hematoablation (25, 40, 41), and the incidence of induced reactivation correlated with latent viral genome load (25, 38, 39). Notably, MI was observed also in mice latently infected with a single-cycle recombinant mCMV unable to reactivate to production of infectious virions because of genetic deletion of glycoprotein L (29). Thus, experimental data do not support the hypothesis of frequent events of productive reactivation being the driver of MI.

However, a modification of the “reactivation hypothesis”, assuming incomplete reactivation under conditions of immune surveillance, remained valid. So, one might argue that inflationary CD8^+^ T cells sense reactivation events by recognizing antigens expressed in the course of reactivation, and terminate the productive viral cycle before the assembly and release of infectious virions (42). The productive viral cycle is characterized by coordinated gene expression defined for all members of the herpesvirus family as a temporal transcription cascade that is divided into three kinetic classes progressing from the immediate-early (IE) phase, to the early (E) phase, and finally to the late (L) phase (43–45). Thus, if the hypothesis applies, the analysis of transcripts in tissues of latently infected mice should be in conformity with coordinated gene expression.

Here we provide evidence in support of an alternative hypothesis for explaining MI, the “stochastic transcription hypothesis” (8) proposing sporadic episodes of transient de-silencing of genes in latent viral genomes in a stochastic fashion, not following the IE-E-L temporal cascade of productive cycle transcription. Notably, only the “stochastic transcription hypothesis” can explain why expression of viral immune evasion genes does not prevent the presentation of MI-driving antigenic peptides.

## Materials and Methods

### Viruses and Mice

Bacterial artificial chromosome (BAC)-cloned virus MW97.01, derived from BAC plasmid pSM3fr (46, 47), is herein referred to as mCMV-WT. Cell culture-derived high titer virus stocks were generated by a standard protocol (48).

Female BALB/c (8-week-old) mice were purchased from Harlan Laboratories and were housed under specified pathogen-free (SPF) conditions in the Translational Animal Research Center (TARC) of the University Medical Center Mainz.

### Experimental HCT and Establishment of Latent mCMV Infection

Syngeneic hematopoietic cell transplantation (HCT) with 9-week-old female BALB/c mice as bone marrow cell (BMC) donors and recipients was described previously (48, 49). In brief, hematoablative conditioning was performed by total-body γ-irradiation with a single dose of 6.5 Gy. At 6 h after irradiation, 5x10^6^ BMC were infused into the tail vein of the recipients, followed by intraplantar infection with 1x10^5^ PFU of mCMV-WT. Organ infectivity was followed up to 4 months post-HCT by a high-sensitivity plaque assay performed under conditions of “centrifugal enhancement of infectivity” [(34, 48) and references therein].

### Quantitation of Latent Viral Genomes in Lung Tissue

To determine the latent viral DNA load in lung tissue of latently infected mice, DNA from the postcaval lobe was extracted with the DNeasy blood and tissue kit (catalog no. 69504; Qiagen, Hilden, Germany) according to the manufacturer’s instructions. Viral and cellular genomes were quantitated in absolute numbers by *M55*-specific and *pthrp*-specific qPCRs normalized to a log_10_-titration of standard plasmid pDrive_gB_PTHrP_Tdy (50, 51).

### *In Vitro* Transcripts

For generation of *in vitro* transcripts, the sequences encompassing the open reading frames (ORFs) M86, M105, M112/E1, and m152 were amplified by PCR with the respective oligonucleotides (**Table S1**) from viral DNA (strain Smith ATCC VR-1399) as template. The resulting products were inserted *via* UA-cloning into the pDrive vector (Qiagen) to generate pDrive-M86, pDrive-M105, pDrive-E1, and pDrive-m152, respectively. For generation of *in vitro* transcripts for the viral ORFs m04 and m06, the respective sequences were amplified by PCR and inserted *via* the HindIII and XmaI restriction site into vector pSP64 Poly(A) (Promega, Madison, WI). All vectors were linearized with EcoRI (ThermoFisher Scientific, Langenselbold, Germany) and used as template for *in vitro* transcription with the MAXIscript SP6/T7 Transcription Kit (catalog no. AM1320, ThermoFisher Scientific). *In vitro* transcripts IE1 and m164 were described previously (52, 53).

### Analysis and Quantitation of Transcripts

Viral transcripts in latently infected lungs were detected by quantitative reverse transcriptase PCR (RT-qPCR) as described previously (50). Briefly, lungs of latently infected HCT recipients were cut into pieces followed by shock-freezing in liquid N2. Total RNA was isolated with the RNeasy Mini Kit (catalog no. 7410, Qiagen) according to the manufacturer’s instructions, including an on-column DNAase I (catalog no. 79254, Qiagen) digestion. Synthesis of cDNA and transcript quantification were performed using 100 ng of RNA per sample and the OneStep RT-PCR Kit (catalog no. 210212, Qiagen). For absolute quantitation, dilution series of specific *in vitro* transcripts served as standards (50). In parallel, cellular β-actin transcripts were quantified for normalization. For primers and probes, see **Table S2**.

### Separation of Lung Endothelial Cells

Endothelial cells (EC) from latently infected mice were sort-purified from single-cell suspensions of lung tissue by cytofluorometric cell sorting. Single-cell suspensions were prepared essentially as described previously (22, 48), though with modifications. In brief, lungs were perfused with PBS supplemented with 10U/ml Heparin (Ratiopharm, Ulm, Germany). Lungs were excised, tracheae, bronchi, and pulmonary lymph nodes were discarded, and the lung lobes were minced. The tissue of 4-5 lungs was digested in 15 ml Gey’s Balanced Salt Solution (GBSS), containing collagenase A (1.6 mg/mL; catalog no. 10 103 586 001, Roche, Mannheim, Germany) and DNase I (50 µg/mL; catalog no. DN-25, Sigma-Merck, Darmstadt, Germany), for 1 h at 37°C with constant stirring. The resulting cell suspension was washed with GBSS, and after lysis of erythrocytes washed again with GBSS. After blocking of Fc receptors with CD16/CD32 monoclonal antibody (mAb), cells were labeled with R-phycoerythrin (R-PE)-conjugated rat anti-mouse CD31 mAb (clone 390, AbD Serotec, Kidlington, United Kingdom). The cell pellet was resuspended in phosphate-buffered saline (PBS) containing 1% FCS, and cells were labeled with fluorescein isothiocyanate (FITC)-conjugated rat anti-mouse mAb CD146 (clone ME-9F1, Miltenyi Biotec, Bergisch Gladbach, Germany). CD31^+^CD146^+^ ECs were isolated at the FACS core facility (IMB Mainz, Germany) by cytofluorometric cell sorting using BD FACS Aria (BD Bioscience, San Jose, CA).

### Cytofluorometric Analyses

Single-cell suspensions were prepared from lung tissue as described (22, 48). Unspecific staining was blocked with unconjugated anti-FcγRII/III antibody (anti-CD16/CD32; clone 2.4G2, BD Pharmingen, Heidelberg, Germany). Cells were specifically stained with the following antibodies for multi-color cytofluorometric analyses: ECD-conjugated anti-CD8α (clone 53-6.7; Beckman Coulter, Krefeld, Germany), FITC-conjugated anti-KLRG1 (clone 2F1; eBioscience, Frankfurt), PE-Cy5-conjugated anti-CD127 (clone A7R34; eBioscience, Frankfurt), and PE-Cy7-conjugated anti-CD62L (clone MEL-14; Beckman Coulter). Phenotypic characterization of peptide-specific CD8^+^ T cells was performed using PE-conjugated dextramers H-2Ld/YPHFMPTNL (IE1), H-2Dd/AGPPRYSRI (m164), and H-2Kd/TYWPVVSDI (M105) (22, 31). H-2Kb/SIINFEKL served as the control for excluding unspecific staining (Immudex, Copenhagen, Denmark). For the analyses, a “live gate” was routinely set on leukocytes in the forward scatter (FSC) versus sideward scatter (SSC) plot. All cytofluorometric analyses were performed with flow cytometer FC500 and CXP analysis software (Beckman Coulter).

### Statistical Calculations

Statistical significance of differences between two independent sets of data was evaluated using the two-sided unpaired *t*-test with Welch’s correction for unequal variances. In the case of genome quantification, log-normally distributed data were log-transformed to enter the *t-*test. Differences were considered as statistically significant for p-values of <0.05 (*), <0.01 (**) and <0.001 (***). Graph Pad Prism 6.04 (Graph Pad Software, San Diego, CA) was used for the calculations. Frequencies of transcriptional events were estimated from the fraction of transcript-negative pieces by using the Poisson distribution function as described (54, 55). Detection limits for viral transcripts and the corresponding 95% confidence intervals (CI) were determined by limiting dilution analysis as described (54–56). The null hypothesis of independent distribution of pairs of gene expression events was evaluated by organizing data in 2x2 contingency tables for calculation by Fisher’s exact probability test (https://www.socscistatistics.com/tests/fisher/default2.aspx) (57). The null hypothesis is not refuted, and thus a correlation not assumed, if p >0.05.

## Results

### Viral Transcription in Latently Infected Lungs Comprises Genes of the Three Kinetic Classes IE-E-L of the Viral Replication Cycle and Declines Over Time

**Figure 1:**
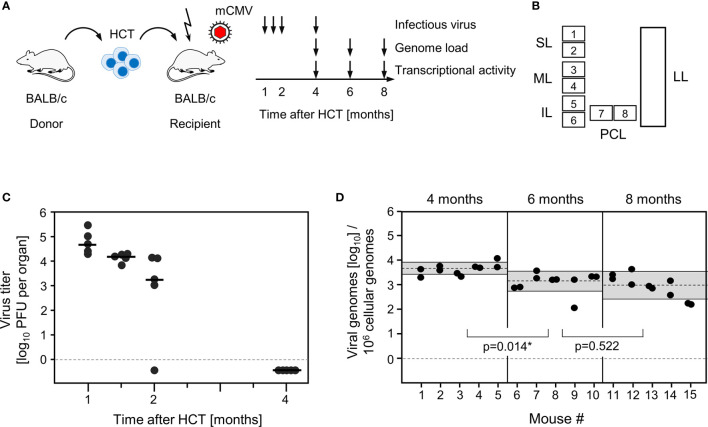
Outline of the model and verification of the establishment of viral latency in the lungs. **(A)** Sketch of the experimental model of syngeneic hematopoietic cell transplantation (HCT) with BALB/c mice as hematopoietic cell donors and recipients, and schedule of assays. The flash symbol indicates hematoablative treatment of the recipients by total-body γ-irradiation with a single dose of 6.5 Gy prior to performing HCT and infection with mCMV. **(B)** Scheme of the lungs in anatomical view with tissue pieces p1-p6 used for quantitation of viral transcripts, p7 and p8 used for quantitation of viral transcripts and latent viral DNA load simultaneously, and the left lung used for cytofluorometric analyses. SL, superior lobe; ML, middle lobe, IL, inferior lobe; PCL, postcaval lobe, LL, left lung. **(C)** Virus titers expressed as plaque-forming units (PFU) per organ, were determined under conditions of centrifugal enhancement of infectivity. Routinely, 1% aliquots of lung homogenate were tested. Negative results were confirmed by plating the homogenate in total to avoid a sampling error. Symbols represent data from individual mice. Median values are marked. **(D)** Latent viral DNA load determined for lung tissue pieces p7 and p8 of the PCLs of mice #1-to-#5 (at 4 months), #6-to-#10 (at 6 months), and #11-to-#15 (at 8 months). Each single symbol represents the mean value from triplicate determinations. The shaded areas indicate the 95% confidence intervals for the log-normally distributed data. (*) Data sets are considered as being significantly different if p < 0.05.

Lungs represent a major organ site of CMV pathogenesis in clinical HCT (reviewed in (58)) and in the mouse model of experimental HCT (59). They were identified as a site of high latent viral DNA load and high risk of reactivation to recurrent infection in mouse models of neonatal infection (25, 38) and infection under conditions of HCT (34, 39). In addition, the phenomenon of MI was originally observed for CD8^+^ T cells in persistent pulmonary infiltrates in the mouse model of HCT and mCMV infection (2, 59). We thus focus here on studying viral transcription and MI in latently infected lungs in the well-established HCT model (**Figures 1A, B**). In accordance with previous work in this model (60), productive viral replication was cleared by 4 months after HCT and infection, based on the high-sensitivity assay of “centrifugal enhancement of infectivity” (34) (**Figure 1C**). Viral genome remained present in the lungs over the entire observation period of 8 months with a statistically significant decline only between 4 months and 6 months (**Figure 1D**). So, the definition of viral latency, namely presence of viral genome in absence of infectious virus (36), is fulfilled in our study, which is a precondition for linking MI to viral latency.

Already at a time before MI was discovered, we showed stochastic activity of mCMV IE-phase gene *ie1* during latent infection of the lungs as a first example of a “transcript expressed in latency” (TEL) [(52), reviewed in (8)], and soon later stochastic and independent expression of genes *ie1* and *ie2* was described (54). These two genes flank the major IE promoter-enhancer and form a bidirectional gene pair governed by independent promoters (61, 62). As gene *ie1* encodes protein IE1/pp89, which contains the antigenic peptide IE1-YPHFMPTNL that is presented by the MHC class-I molecule L^d^ (63), we proposed episodes of antigen presentation during latent infection in absence of completion of the viral productive cycle (52). The IE1 peptide was the first peptide shown to drive MI of CD8^+^CD62L^-^ T effector-memory cells (TEM) (2). A role for IE1-specific CD8^+^ T cells in immune sensing and surveillance of latent mCMV infection was proposed in the original report on antigenicity of IE proteins (64), and an antigenicity loss mutant coding for peptide IE1-YPHFMPTNA provided evidence for this (42). Only recently, transcripts coding for the second MI-driving peptide of mCMV in the *H-2^d^* haplotype, namely peptide m164-AGPPRYSRI presented by the MHC class-I molecule D^d^ (3), were detected in latently infected lungs (56). To our knowledge, in other mouse models of MI, such as MI in the *H-2^b^* haplotype, transcription during viral latency of genes coding for MI-driving antigenic peptides has not been studied.

**Figure 2:**
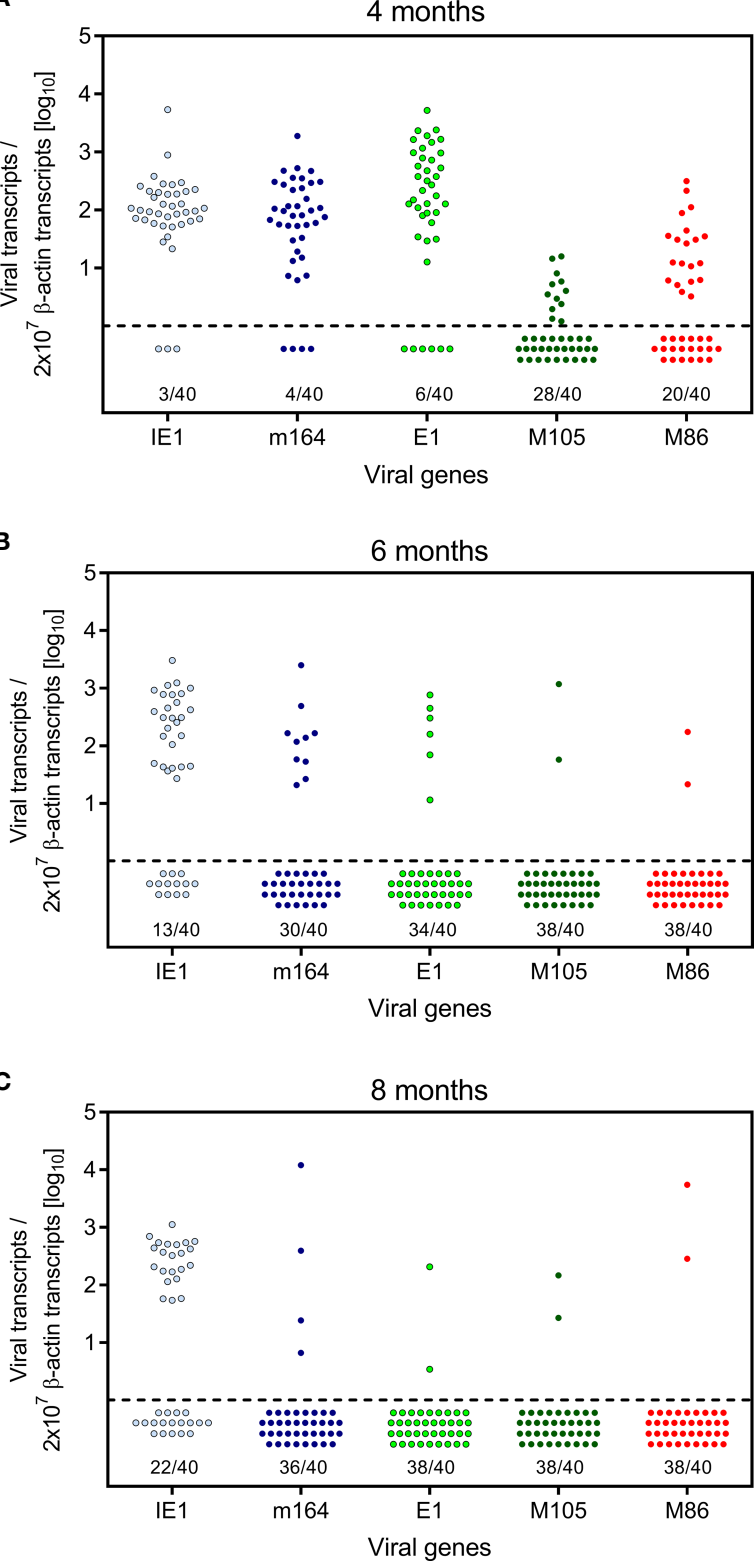
Kinetics of viral transcriptional activity in latently infected lungs. Transcripts from the indicated IE, E, and L phase-marker genes were quantitated by RT-qPCR at **(A)** 4 months, **(B)** 6 months, and **(C)** 8 months after HCT and infection. Symbols represent data from lung tissue pieces p1-to-p8 of 5 mice, that is altogether 40 pieces per time of analysis. Pieces negative in the respective RT-qPCR are shown below the dashed line. The proportion F(0) of negative samples is indicated.

Here we have addressed the question if transcription during latency follows the coordinated and temporal gene expression cascade of the productive viral cycle, characterized by directed IE-E-L phase progression (see the Introduction). For a clonal analysis, we used the established method of quantitating viral transcripts in individual tissue pieces of latently infected lungs (41, 42, 51, 52, 54). We chose transcripts to represent viral proteins characterizing the three kinetic phases. The MI-driving protein IE1 represents the IE phase, the MI-driving protein m164 is expressed in both the IE and the E phase (56), the E phase proteins E1 (65–67) and M105 (68) are both critically involved in viral DNA replication, and the L phase protein M86 is the major capsid protein (MCP) essential for virion structure and assembly (69). We quantitated transcripts by RT-qPCR at 4, 6, and 8 months after HCT and infection. The analysis was performed for 5 latently infected mice per time and for 8 lung tissue pieces p1-p8 per lung, derived from the three lobes of the right lung and the postcaval lobe (for a scheme, see **Figure 1B**). Thus, 40 pieces altogether were analysed per time. The detection limits for the 5 phase-marker transcripts as well as for the transcript of the house-keeping gene standard *β-actin* in the respective RT-qPCRs were determined by limiting dilution analysis of synthetic transcripts, and were found to be comparable with overlapping 95% confidence limits (55) (**Figure S1**). At a glance, all phase-marker genes were found to be expressed in latently infected lungs, although also transcript-negative pieces existed for each of them (**Figures 2A–C**). This already indicated critical gaps in the IE-E-L cascade. The overall transcriptional activity declined over time, with the strongest recession between 4 and 6 months (**Figures 2A, B**), corresponding to a loss of latent viral genomes in this period (**Figure 1D**).

### Viral Transcription in Latently Infected Lungs Follows Stochastic Patterns Incompatible With the Temporal Gene Expression Cascade During Productive Reactivation

**Figure 3:**
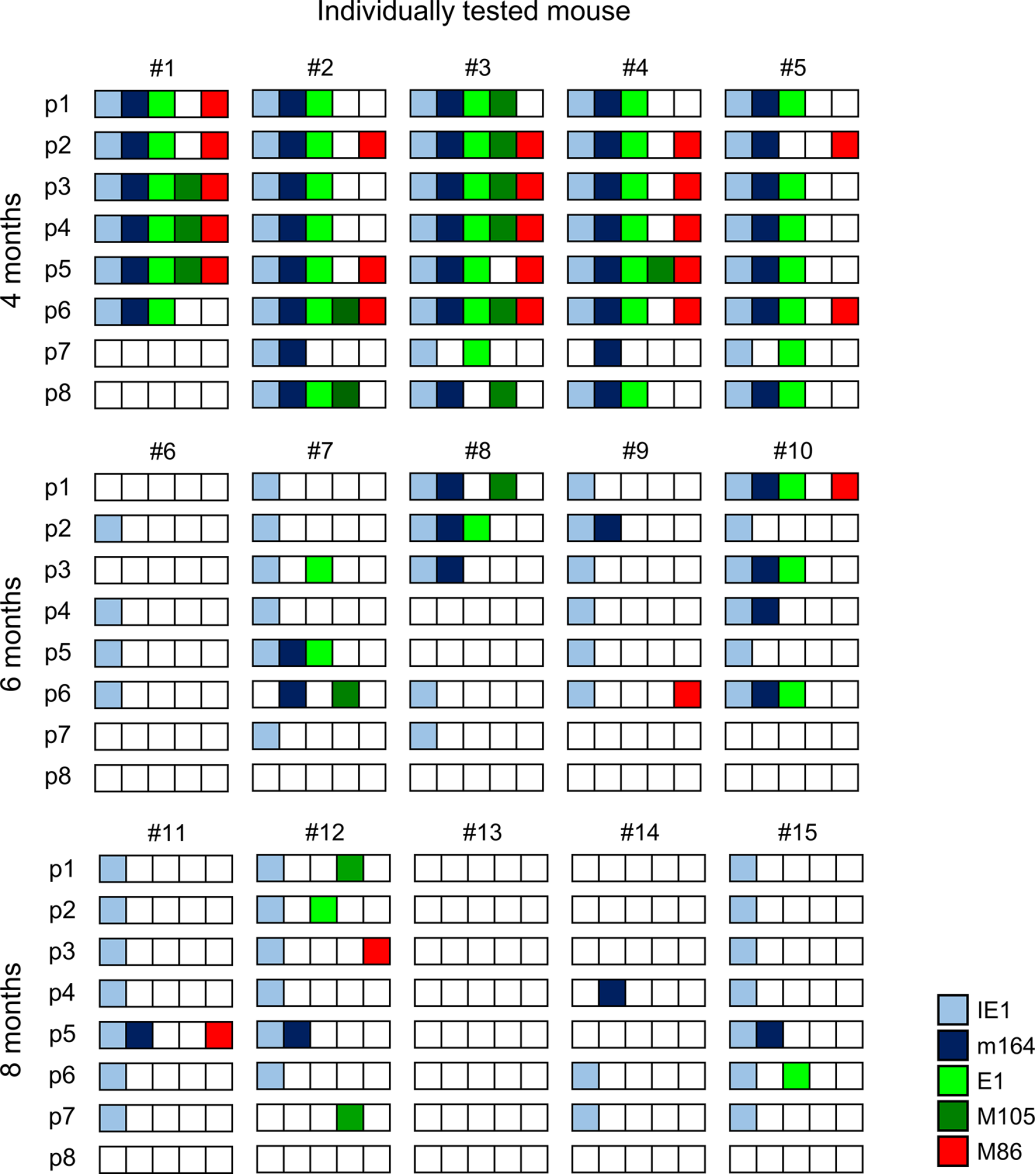
Stochastic gene expression patterns in latently infected lungs. The quantitative gene expression data shown in **Figure 2** were categorized into positive or negative for transcripts from the respective viral gene and were assigned to the lung tissue pieces p1-to-p8 of mice #1-to-#15. Boxes negative for the respective transcripts are left blank, boxes positive for the respective transcripts are shown color-coded as specified in the internal legend.

The quantitative expression data (**Figure 2**) were categorized into tissue pieces being positive or negative for TEL from the five chosen phase-marker genes, and the resulting contextual expression patterns are shown for the 40 lung tissue pieces per time of analysis to reveal the genes expressed in the individual pieces (**Figure 3**). At 4 months after HCT and infection, the overall high transcriptional activity prevented a formal exclusion of productive infection in the 9 out of 40 pieces (#1p3/4/5, #2p6, #3p2/3/4/6, and #4p5) in which all of the five chosen phase-marker genes were found to be expressed. However, as mCMV has a coding capacity of about 200 open readings frames specifying many more essential E and L phase genes (70, 71), these 9 pieces most likely have unidentified other critical gaps in the gene expression cascade. The stochastic mode of gene expression without completion of the productive cycle becomes more evident at 6 months and 8 months when the overall transcriptional activity has declined. Notably, there even existed pieces in which the late gene *M86* was expressed, although the E phase genes *e1* and *M105*, which are essential for progression to the L phase, were both not expressed. Examples are pieces #9p6, #11p5, and #12p3.

**Figure 4:**
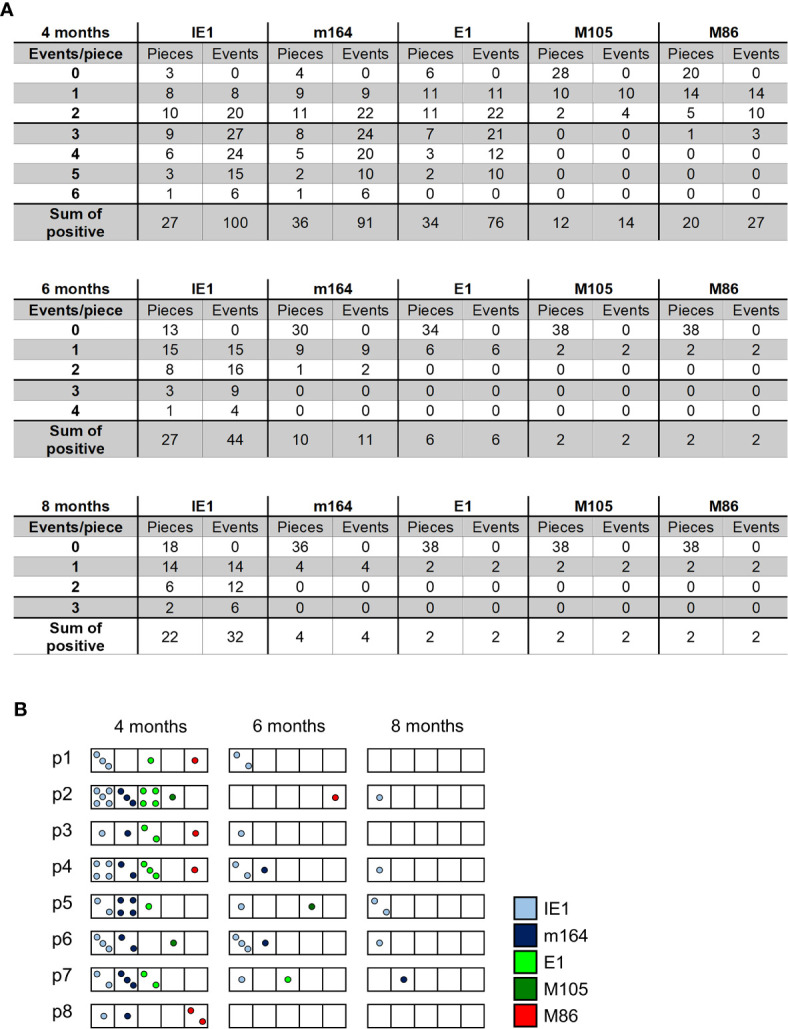
Quantitation of transcriptional episodes in latently infected lungs. **(A)** Poisson distribution analysis of clonality. Based on the fraction of tissue pieces negative for transcripts from the respective viral gene (**Figure 2**), the Poisson distribution parameter λ = - lnF(0) allows the calculation of the numbers of clonal F(n=1), biclonal F(n=2) and oligoclonal F(n >2) transcription events according to the formula F(n) = λ/n x F(n-1). **(B)** Illustration of clonality for a “statistical lung”, representing the average of lungs derived from 5 mice per time of analysis.

The existence of negative pieces for any of the five phase-marker genes indicates stochastic events that follow the Poisson distribution function, from which one can calculate the number of transcription events (54). This number is higher than the number of positive pieces, because a positive piece necessarily reflects at least one clonal transcription event but may comprise also more than one transcription event. The fraction of negative pieces F(0) (**Figures 2A–C**) defines the Poisson distribution parameter lambda (λ) = - lnF(0) and allows to calculate the fraction F(n) of tissue pieces with 1,2, …n transcription events according to the formula F(n) = λ/n x F(n-1) (41, 42, 51, 54) (**Figure 4A**). For an illustration, the occupancies of tissue pieces with TEL events are shown for 8 pieces of statistically averaged lungs by down-extrapolating data from 40 pieces from five mice per time of analysis (**Figure 4B**). This illustrates that TEL are mostly of clonal origin at 6 and 8 months, with the exception of IE1 for which biclonal and triclonal transcription existed.

### Memory Inflation and Deflation Reflect Preceding Events of Transcription During Viral Latency

**Figure 5:**
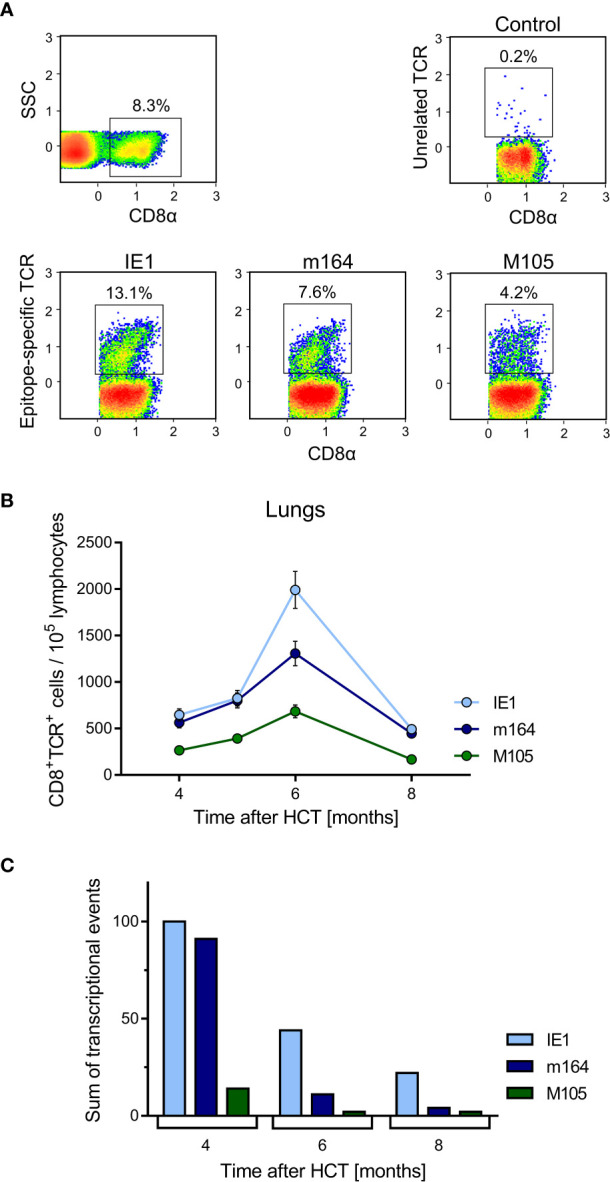
Time course of MI of viral epitope-specific CD8^+^ T cells in the lungs. **(A)** Gating strategy for the cytofluorometric quantitation of pulmonary CD8^+^ T cells expressing T-cell receptors specific for the pMHC-I complexes IE1-L^d^, m164-D^d^, and M105-K^d^. Control, PE-conjugated pMHC-I dextramer H-2Kb/SIINFEKL. SSC, sideward scatter. Data refer to 6 months after HCT and infection. **(B)** Response kinetics of viral epitope-specific CD8^+^ T cells isolated from pulmonary infiltrates of the LL at the indicated times after HCT and infection. Note that, so far, antigenic peptides are not identified for proteins E1 and M86. Shown are median values and range for five mice per time of analysis. **(C)** Corresponding total numbers of transcriptional events as determined in SL, ML, IL, and PCL (recall **Figure 4A**).

**Figure 6:**
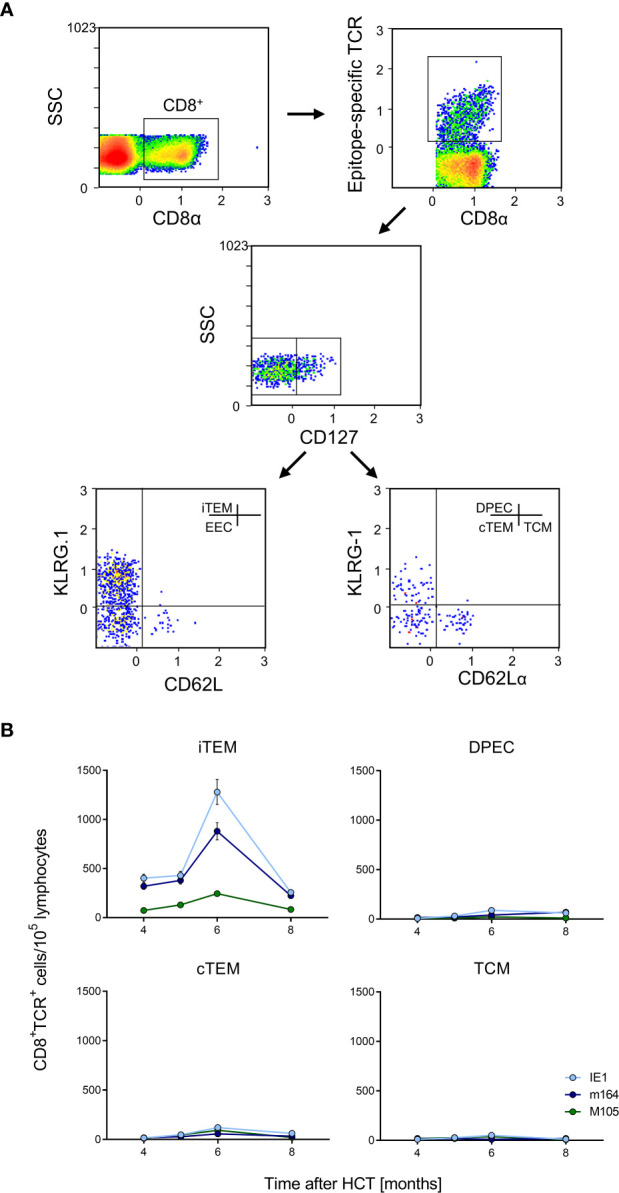
Identification of iTEM as the “inflationary” subset of viral epitope-specific CD8^+^ T cells in the lungs. **(A)** Gating strategy for the cytofluorometric quantitation of viral epitope-specific KLRG1^+^CD62L^-^CD127^-^ inflationary effector-memory cells (iTEM), KLRG1^-^CD62L^-^CD127^-^ early effector cells (EEC), KLRG1^+^CD62L^-^CD127^+^ double-positive effector cells (DPEC), KLRG1^-^CD62L^-^CD127^+^ conventional effector-memory cells (cTEM), and KLRG1^-^CD62L^+^CD127^+^ central memory cells (TCM). SSC, sideward-scatter. Data refer to IE1-specific cells measured at 6 months after HCT and infection. **(B)** Response kinetics of the indicated subsets of viral epitope-specific CD8^+^ T cells isolated from pulmonary infiltrates of the LL at the indicated times after HCT and infection. Shown are median values and range for five mice per time of analysis.

In an attempt to relate MI to transcription of epitope-encoding genes, we used the left lung of the mice, corresponding to the TEL analyses performed with the three lobes of the right lung and the postcaval lobe (for a scheme, see **Figure 1B**), to isolate lung-infiltrating lymphocytes for cytofluorometric analyses (**Figures 5**, **6**). We quantitated lung-infiltrate CD8^+^ T-lymphocytes specific for the known antigenic peptides IE1, m164, and M105 in the *H-2^d^* haplotype (reviewed in (72)), shown exemplarily for 6 months (**Figure 5A**). The full kinetics of frequencies, normalized to lung-infiltrating lymphocytes (**Figure 5B**), is compared to TEL activity in terms of transcriptional events determined in parallel in the same cohort of mice (**Figures 4A** and **5C**). As the bottom-line message, MI peaking at 6 months is preceded by high TEL activity, and the deflation seen at 8 months is preceded by a decline in TEL activity.

Latent mCMV genomes reside in non-hematopoietic tissue cells in organs, specifically in endothelial cell (EC) types [(73), reviewed in (8, 35)], including CD31^+^CD146^+^ EC in the lungs (**Figure S2**). Previous work in chimera models has shown that MI depends on antigen presentation by non-hematopoietic cells (60, 74). In models of MI after systemic infections that result in a high latent virus genome load in organs, the expanding CD8^+^ T-cell pool is made up primarily of inflationary effector-memory T cells (iTEM) characterized by the cell surface phenotype KLRG1^+^CD62L^-^ (20, 60, 74). In contrast, these cells decline over time after local primary infection that leads to an only low latent virus genome load in organs (22). Here we have included the marker molecule CD127 (IL-7Rα) to further distinguish between KLRG1^+^CD127^-^ iTEM and KLRG1^+^CD127^+^ double-positive effector cells (DPEC) (75, 76) as well as KLRG^-^CD127^+^ conventional effector-memory T cells (cTEM) within CD8^+^CD62L^-^ cells (**Figure 6A**). As we have shown previously that the majority of cells of long-term cytolytic T-lymphocyte lines (CTLL) propagated in cell culture assume DPEC phenotype (72), we surmised that repetitive antigen restimulation by latently infected cells might generate CTLL *in vivo*. However, the kinetics revealed an MI predominantly made up by iTEM with just minimal contributions from DPEC and cTEM. As expected, the pool of KLRG1^-^CD127^+^CD62L^+^ central memory T cells (TCM) does not expand at the non-lymphoid site of lung tissue (**Figure 6B**). A triple-negative population of KLRG1^-^CD127^-^CD62L^-^ cells, discussed as representing early effector cells (EEC) (75, 76), is not further considered here as it does not participate in MI (data not shown).

While our focus was here on the lungs as the most prominent site of CMV pathogenesis in HCT recipients, EC or EC-related cells are cellular sites of latent mCMV infection also in other organs, including the spleen (reviewed in (35)), and thus likely contribute to MI. As a central lymphoid organ and organ site of mCMV latency, the spleen can harbor recirculating T cells that have received an antigen signal during the patrolling of non-lymphoid tissue sites, but can also provide an antigen signal locally. We have therefore studied MI in the spleen in parallel in the same experiment for which lung data are shown above (**Figure S3**, corresponding to **Figures 5**, **6**). In essence, like in the lungs, MI in the population of CD8^+^ T cells is predominantly made up of iTEM with the same hierarchy of viral epitopes and similar kinetics, though with some distinctive differences. Specifically, the relative decline in numbers of iTEM between 6 months and 8 months was less for epitopes IE1 and m164 compared to the lungs, whereas the number of iTEM specific for epitope M105 was even slightly increasing. Notably, TCM stayed at low-level throughout the observation time. Based on previous findings on secondary iTEM pool contraction at late times due to exhaustion of frequently re-sensitized high-avidity cells (22), we speculate that re-stimulation by local TEL activity is generally less frequent in the spleen compared to the lungs, and is particularly rare for epitope M105 for which the iTEM pool continued to expand instead of contract.

### Stochasticity of Viral Gene Expression During Latency Allows MI by Avoiding Immune Evasion

CMVs express immune evasion proteins that interfere with cell surface trafficking of peptide-loaded MHC class-I (pMHC-I) complexes in the MHC class-I pathway of antigen processing and presentation (reviewed in (77, 78)). In the case of mCMV, three “viral regulators of antigen presentation” (vRAP) operate in the E phase. The negative vRAP m06/gp48 (79, 80) and the positive vRAP m04/gp34 (81–83) compete for pMHC-I cargo in post-Golgi network sorting to the lysosome and the cell surface, respectively. They thus oppose each other when co-expressed (84). In consequence, during productive infection when both are expressed, immune evasion is primarily determined by the negative vRAP m152/gp40 that traps pMHC-I complexes in a *cis*-Golgi/ER intermediate-Golgi compartment (85, 86).

Given the overall low transcriptional activity of viral epitope-encoding genes during latency, one wonders why the mechanisms of immune evasion do not interfere with antigen presentation and thus do not prevent MI. If the “reactivation hypothesis” applies, reactivation originating from viral genomes in a latently infected cell would proceed along the programmed IE-E-L phase progression (see the Introduction) and must inevitably reach the point at which the E-phase vRAP m152/gp40 is expressed to interfere with pMHC-I cell surface presentation and thus also with MI. If, however, the “stochastic transcription hypothesis” applies, epitope-encoding viral genes and immune evasion molecule-encoding genes are not necessarily expressed in the same cell and thus do not meet each other.

**Figure 7:**
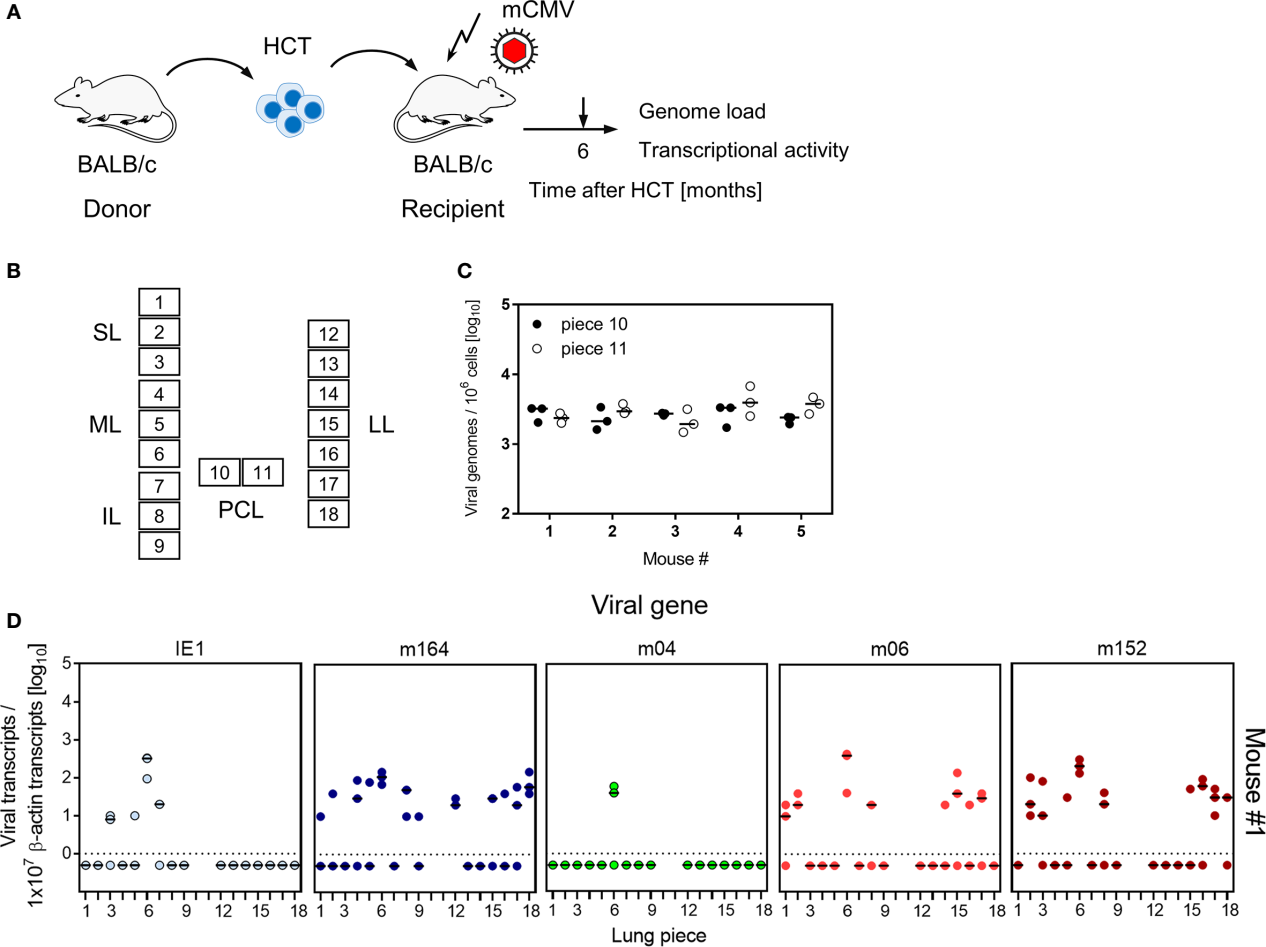
Transcriptional activity of MI-driving and of vRAP-encoding genes in latently infected lungs. **(A)** Sketch of the experimental model of HCT and infection (for more explanation, see **Figure 1**). **(B)** Scheme of the lungs in anatomical view with tissue pieces p1-to-p9 and p12-to-p18 used for quantitation of viral transcripts. Pieces p10 and p11 were used to determine the latent viral DNA load. SL, superior lobe; ML, middle lobe, IL, inferior lobe; PCL, postcaval lobe, LL, left lung. **(C)** Viral DNA load determined in triplicates. The median values are marked. **(D)** Quantitation of transcripts from the indicated genes shown exemplarily for lung tissue pieces from mouse #1. Mice #2-to-#5 were analysed accordingly. Symbols represent triplicate measurements. The median values are marked.

To decide between these two hypotheses, we studied transcription of MI-driving genes and of vRAP-encoding genes in latently infected lungs of five mice at 6 months after HCT and primary infection (**Figure 7**, for the experimental protocol of HCT, see **Figure 7A**). To enhance statistical resolution, lungs were subdivided into 18 pieces (**Figure 7B**). The two pieces of the postcaval lobe, p10 and p11, served to determine the latent viral DNA load (**Figure 7C**). Pieces p1-p9 of the three lobes of the right lung and pieces p12-p18 of the left lung, altogether 80 pieces of the lungs of 5 mice, were used to detect transcripts encoding MI-driving proteins IE1 and m164, as well as transcripts encoding vRAPs m04, m06, and m152 (**Figure 7D**).

**Figure 8:**
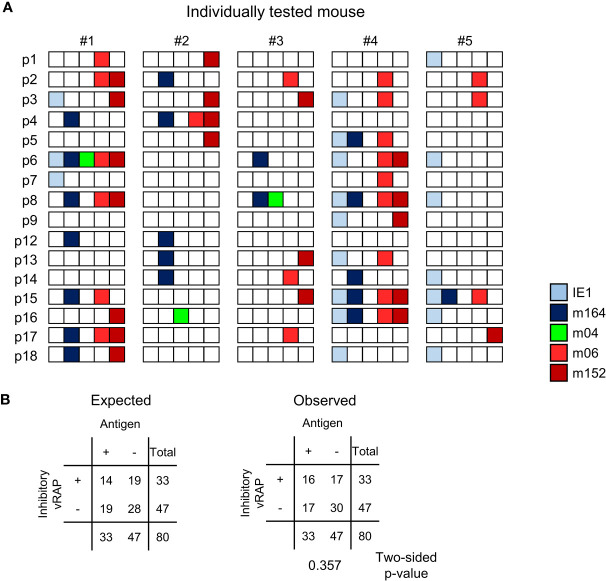
Stochastic gene expression patterns of MI-driving and of vRAP-encoding genes in latently infected lungs. **(A)** The quantitative gene expression data, shown in **Figure 7D** exemplarily for mouse #1, were categorized for all five mice into positive or negative for transcripts from the respective viral gene and were assigned to the lung tissue pieces p1-to-p9 and p12-to-p18 of mice #1-to-#5. Boxes negative for the respective transcripts are left blank, boxes positive for the respective transcripts are shown color-coded as specified in the internal legend. **(B)** Expected and observed 2 x 2 contingency tables for the expression of MI-driving antigens (IE1 and m164 combined) and of immunoevasive vRAPs (m06 and m152 combined) for testing the null hypothesis of independent distribution with Fisher’s Exact Probability Test. The null hypothesis is accepted for p >0.05.

At a glance, for each of the lungs of the 5 mice tested, the summarized expression patterns reveal tissue pieces in which either or both of the MI-driving antigens IE1 and m164 are expressed in absence of both inhibitory vRAPs m06 and m152 (**Figure 8A**). Examples are pieces #1p4/7/12, #2p2/12/13/14, #3p6/8, #4p14/18, and #5p1/6/8/14/16/18. The positive vRAP m04 was rarely expressed, specifically only in pieces #2p16 and #3p8, so that it can be neglected in this particular experiment. Altogether, there apparently existed quite a number of tissue pieces in which absence of immune evasion allowed the presentation of MI-driving antigenic peptides.

Moreover, inhibitory vRAPs can only operate when expressed in the same cell that expresses a MI-driving antigen. They cannot inhibit antigen presentation in a neighboring cell. In consequence, double occupancy of a tissue piece with MI-driving transcription events and immune evasion-mediating transcription events does not necessarily imply that immune evasion is operative. If cases of co-expression in the same cell were frequent, the number of pieces simultaneously positive for antigen-encoding and inhibitory vRAP-encoding transcripts should be higher than expected by the null hypothesis of independent distribution of these transcriptional events, that is, expression in different cells. As revealed by Fisher’s Exact Probability Test comparing the observed 2x2 contingency table with the one expected for independent distribution (57), the hypothesis of independence was accepted with p >0.05 (**Figure 8B**).

In conclusion, viral immune evasion does not prevent MI-driving antigen presentation, because antigens and inhibitory vRAPs are rarely co-expressed in the same cell.

## Discussion

It was the aim of our study to contribute to the open question of how MI-driving antigens are provided during viral latency, a state defined by absence of infectious virus despite presence of viral genomes in latently infected cells, from which reactivation of the full transcriptional program to productive infection can be re-initiated [for reviews, see (35, 36)]. Roizman’s definition of herpesvirus latency, originally proposed for alpha-herpesviruses (36), has long been disputed with the alternative hypothesis of “low-level persistent infection” below the detection limit of assays for infectious virus. Obviously, low-level persistent infection would elegantly explain sustained provision of antigens for driving MI. However, epigenetic silencing of essential genes of the productive/lytic viral cycle and latency-specific patterns of latency-associated transcription argued for the existence of true molecular latency of the beta-herpesvirus hCMV in hematopoietic progenitor cells committed to the myeloid lineage [for reviews, see (35, 87)]. Specifically, as IE genes code for essential transactivator proteins in the viral program of replication, absence of lytic cycle IE transcripts long served as molecular evidence for latency. In turn, presence of lytic cycle IE transcripts was taken as indicating productive infection, though it is hardly possible to draw a clear distinction between a continual ‘persistent’ infection and frequent episodes of reactivation from latency that mimic persistence the better the shorter the intervals are. On the organismal level, latent infection and truly persistent or intermittent reactivated productive infections can co-exist compartmentalized to different cell types and organs. For instance, after acute mCMV infection, virus replication persists for some time in glandular epithelial cells of salivary glands when latency is already established in cells of other organs (25), such as in liver sinusoidal endothelial cells (LSECs) (73). Likewise, experimentally provoked reactivation of latent mCMV in organs was found to be a stochastic process that can take place in any one organ that harbors latent viral genomes, while other organs stay latently infected (25, 26). Similarly, during clinical hCMV latency, children can shed low levels of virus from infected epithelial cells in the salivary glands or kidneys for months to years, while, at the same time, infection is already latent in hematopoietic myeloid lineage progenitor cells [for reviews, see (26, 88)].

Epigenetic switches are thought to determine the transition of the viral genome into and out of latency [for reviews, see (87, 89)]. The binary view of viral gene silencing during molecular latency and coordinated de-silencing upon productive reactivation has been challenged for hCMV by highly sensitive assays that detected low levels of viral transcripts from each of the three kinetic gene classes IE, E, and L in latently infected myeloid lineage hematopoietic cells [reviewed in (90)]. This led Collins-McMillen and Goodrum to propose an equilibrium between “true latency” characterized by a pattern of restricted latency-associated transcription and “dynamic latency” where IE, E, and L genes of the lytic program are expressed sporadically not following the canonical temporal IE-E-L cascade of productive reactivation (90). More recent work on the transcriptome of latent hCMV determined by single-cell RNA sequencing (scRNAseq) arrived at the conclusion that latently infected CD34^+^ hematopoietic progenitor cells (HPCs) as well as CD14^+^ monocytes express a broad spectrum of canonical viral lytic cycle genes at a low level [(91), reviewed in (92)]. Based on these findings, cells latently infected with hCMV almost certainly express antigen-encoding viral genes and thus could potentially present antigenic peptides driving MI. However, a retrospect on studies of the immune response to hCMV revealed only limited evidence supportive of MI occurring in humans (23). One may speculate that missing or inhibited MI in latently infected humans may relate to the virally encoded interleukin-10, a form of which is expressed in cells latently infected with hCMV [(93, 94), reviewed in (95)]. Alternatively, in view of the broad spectrum of transcripts revealed by scRNAseq in latently infected myeloid lineage hematopoietic cells, expression of immune evasion genes interfering with the MHC/HLA class-I pathway of antigen presentation (77) might prevent MI.

In contrast to hCMV, the predominant cell types in which mCMV latency is established are not myeloid lineage hematopoietic cells but are EC, as shown for LSECs in the liver (73) and for EC of the capillary bed of the lungs [this report]. In accordance with viral latency in ECs, MI of KLRG1^+^CD62L^-^ iTEM during latency has been shown to be driven by non-hematopoietic cells (60, 74), and the finding that iTEM proliferate in response to viral antigen presented by cells that are accessible to the blood supply (96) is compatible with antigen presentation by latently-infected EC of the lung microvasculature. Notably, our previous work has shown that genes coding for the MI-driving antigenic peptides IE1 and m164 are expressed in latently infected lungs (52, 54, 56).

**Figure 9:**
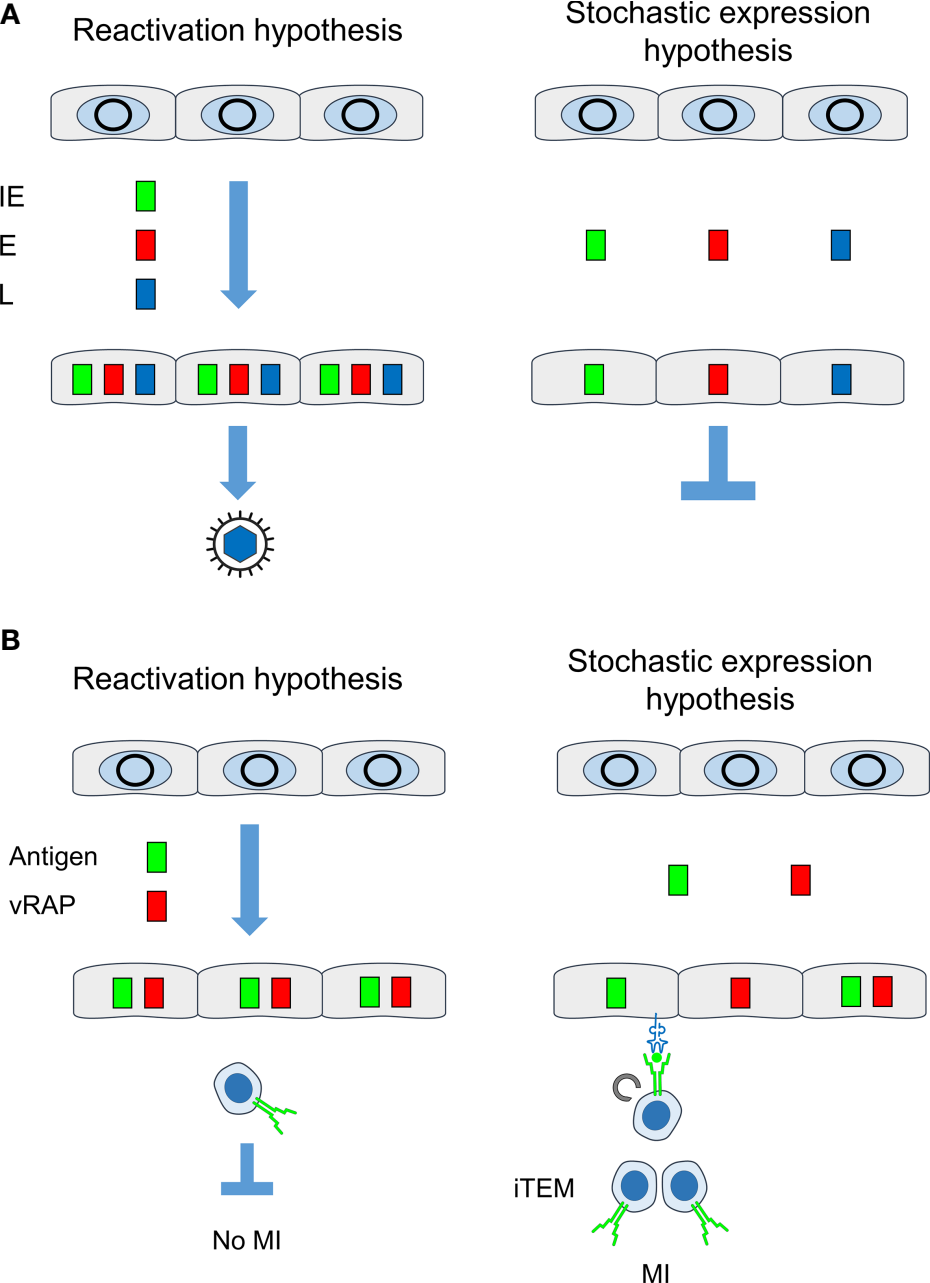
Comparison between “reactivation hypothesis” and “stochastic expression hypothesis”. **(A)** Consequences on the temporal order of viral gene expression and on recurrence of infectious virus. **(B)** Consequences on antigen presentation for driving MI. IE, immediate-early phase or protein; E, early phase or protein; L, late phase or protein; vRAP, (negative) viral regulator of antigen presentation, iTEM, inflationary effector-memory CD8^+^ T cells; MI, memory inflation. Grey circles, silenced circularized viral genomes in the nucleus of latently infected non-hematopoietic tissue cells. Color-coded boxes: viral transcripts and proteins. Receptor symbols on tissue cells and on iTEM represent antigenic peptide-presenting MHC class-I molecules and T-cell receptors, respectively.

It was the aim of our study to decide between two models of MI-driving viral gene expression during latency (**Figure 9A**). The “reactivation hypothesis” proposes episodes of productive virus reactivation characterized by progression of the canonical IE-E-L gene expression cascade within a cell. In contrast, the “stochastic expression hypothesis” proposes stochastic events of viral gene de-silencing that can also generate transcripts of the three kinetic classes IE, E, and L, although not in the temporal order and not necessarily all in one cell. In both models, high latent viral genome load favors high transcriptional activity, because the number of viral genomic DNA molecules determines the probability of productive cycle reactivation as well as of stochastic gene de-silencing. In accordance with this, we found here that the loss of latent viral genomes between 4 and 6 months after infection led to a drop in the transcriptional activity.

Previous work by Snyder and colleagues (29) has shown MI after high-dose systemic infection with a single-cycle recombinant mCMV lacking the essential glycoprotein L. This finding implies that MI does not depend on release of infectious virus and thus modulates the “reactivation hypothesis” in the sense that completion of the lytic cycle is not a demand for driving MI. The interpretation of MI not being driven by recurrence of infectious virus is also strongly supported by the already discussed finding of MI depending on direct antigen presentation by latently infected non-hematopoietic tissue cells (60, 74). This is in accordance with the observation that MI-inducing epitopes do not depend on the immunoproteasome for antigen processing (97). In contrast, virus released after productive reactivation, like virus produced during acute infection (60), should involve hematopoietic lineage antigen-presenting cells, such as dendritic cells, which constitutively express the immunoproteasome.

The conclusion that MI does not depend on productive reactivation was still compatible with the assumption of a canonical IE-E-L gene expression cascade of non-productive reactivation interrupted at a stage before virion assembly and release. However, our data showing stochastic expression patterns of genes of the three kinetic classes falsify the “reactivation hypothesis” and, instead, strongly support the “stochastic expression hypothesis”. Intriguingly, gaps in the expression patterns for essential transcripts provide an immediate explanation for maintenance of latency despite expression of genes of all three kinetic classes. Note that experimentally induced reactivation does, in fact, follow the canonical IE-E-L gene expression cascade and proceeds to the production of infectious virus (41).

It is important to understand that stochastic expression of viral genes during latency means that epigenetic switches between viral chromatin opening and closing, and thus between gene desilencing and silencing, respectively, can be described by the Poisson distribution function, which allows us to calculate the frequency of transcription events at a certain time (54). It is understood that off-on-off states generate expression patterns that fluctuate in the time course, so that what we observe are snapshots. Stochastic expression, however, is not the same as spontaneous expression. As we have shown previously, TNFα signaling to the major immediate-early enhancer of mCMV enhances the transcription from gene *ie1*, which encodes the IE1 protein and MI-driving antigenic peptide (51, 61). In accordance with the stochastic nature of enhancer action (98), this enhancement was caused by increasing the frequency of still stochastic transcription events (51, 61). It is current understanding that signaling increases the probability of transcription initiation, rather than the duration of transcription once it is initiated, although one can recognize the stochastic nature of gene activity only when on-states are rare, as it is the case in viral latency. The frequencies of on-states, as we saw them in the stochastic gene expression patterns, differ significantly between different viral genes (see the numbers of transcription events in **Figure 4A**). As an extreme example, m04 was rarely expressed (**Figure 8**). These differences likely relate to different promoter activities, although experimental proof is pending. The finding that IE1 transcription stands out is reasonably explained by the fact that a strong transcriptional enhancer regulates it. Interestingly, Smith and colleagues recently presented data indicating that stochastic encounters with antigen account for the clonal dynamics during MI (99). Our data provide a molecular explanation for these immunological findings, namely that stochastic encounters of inflationary iTEM with antigen are based on stochastic expression of the corresponding viral genes during latency.

IE1 is the prototype of an MI-driving antigenic peptide (2–4), and this likely relates to the high frequency of transcription during mCMV latency, which corresponds to frequent presentation of IE1 peptide-L^d^ complexes on the surface of latently infected cells for re-stimulating cognate iTEM. At first glance, it may surprise that one of our experiments by chance picked up a case with overall low IE1 transcriptional activity and even absence of IE1 transcripts in 2 out of 5 mice tested individually (**Figure 8**). This brings us to consider two aspects: (i) Transcription patterns represent just snapshots, whereas the iTEM pool “samples” preceding transcription events and memorizes antigen presentation over longer periods. This explains the phase shift between a high rate of transcription at 4 months and the peak number of iTEM at 6 months when transcriptional activity had already declined (compare **Figures 3**, **4** with **Figures 5**, **6**). (ii) iTEM sense antigen presentation while patrolling in tissues for immune surveillance, and then terminate viral gene expression by their effector functions. We concluded this previously from a low frequency of IE1 transcription events in lungs during latent infection with virus mCMV-YPHFMPTNL, which expresses the epitope, and a high frequency after infection with the L9A epitope loss mutant mCMV-YPHFMPTNA (42). Thus, low transcriptional activity might reflect a high level of iTEM activity.

Stochastic expression patterns also help us to understand why reduction of transcription by immune sensing of latently infected cells is epitope-selective. If, for instance, IE1 and m164 were co-expressed in the same cells due to coordinated gene expression during productive reactivation, IE1-specific iTEM should not only reduce IE1 but also m164 transcription events. Apparently, this was not the case (**Figures 7**, **8**). Finally, one should keep in mind that transcriptional activity of epitope-encoding viral genes during latency is a prime condition for MI to occur, but other parameters represent bottlenecks. These include the amount and stability of the antigenic protein (for a recent review, see (100)), the efficacy of its proteasomal processing and the affinity with which the peptide binds to the presenting MHC-I molecule (42, 101), and the functional avidity of the tissue-patrolling CD8^+^ T cells (22).

Our data also offer an elegant answer to the so far pending question of why viral immune evasion proteins/vRAPs do not prevent MI, although they strongly reduce cell surface presentation of pMHC-I complexes (78, 102). Very early work on the role of immune evasion in MI by Gold and colleagues (103) arrived at the conclusion that interference with antigen presentation has little effect on the size of the iTEM pool. For quite some time, this correct finding was mistaken as an evidence against an *in vivo* relevance of immune evasion in general, although soon thereafter a crucial role of immune evasion in the effector phase of the antiviral CD8^+^ T-cell response was demonstrated in many models (for a review, see (104)). Recently, it has been shown that immune evasion is the reason for a failure in preventing lethal virus spread and histopathology in mouse models of allogeneic HCT and CMV infection (105, 106).

This apparent discrepancy is now explained by the stochastic expression of MI-driving genes and immune evasion genes during viral latency. Whereas every cell in which antigens are expressed in the course of reactivation inevitably also reaches the point at which vRAPs become expressed, stochastic gene expression rarely leads to a “by chance co-expression” of MI-driving antigens and vRAPs in the same cell (**Figure 9B**). In consequence, there always exist latently infected cells in which the presentation of MI-driving antigenic peptides is not inhibited by immune evasion.

In an attempt to provide a mathematical model, Gabel and colleagues (37) studied the dynamics of MI in individual mice and found that curve fitting is best when intervals between iTEM re-stimulations are short enough to level the oscillation between expansion and contraction of the iTEM pool. With this understanding, the authors proposed frequent episodes of productive virus reactivation with bursts of virus release providing the antigen for frequent re-stimulation of iTEM. Such a view on MI is, however, incompatible with experimental data showing that productive reactivation is exceedingly rare in latently infected immunocompetent mice (34, 41, 42, 51) and is also incompatible with critical gaps in viral gene expression [this report] as well as with MI induced by the single-cycle virus mutant (29). Instead, the mathematical modeling of MI is in perfect accordance with an almost continuous re-stimulation of iTEM by antigenic peptides derived from episodes of stochastic gene expression during viral latency.

Overall, our data provide reasonable evidence to conclude that stochastic gene expression during viral latency is the viral driver of MI.

## Data Availability Statement

The original contributions presented in the study are included in the article/**Supplementary Material**. Further inquiries can be directed to the corresponding author.

## Ethics Statement

The animal study was reviewed and approved by the ethics committee of the ‘Landesuntersuchungsamt Rheinland-Pfalz’ according to German federal law §8 Abs. 1 TierSchG (animal protection law), permission numbers 177-07/G 10-1-052 and 177-07/G14-1-015.

## Author Contributions

MR and NL are responsible for conception and design of the study, analysis, and interpretation of the data. MG, AR, KF, CS, and NL conducted the work and analysed the data. MR wrote the first draft of the manuscript. NL wrote sections of the manuscript. All authors contributed to the article and approved the submitted version.

## Funding

This work was supported by the Deutsche Forschungsgemeinschaft (DFG), SFB490, individual project E4 “Antigen presentation under the influence of murine cytomegalovirus immune evasion genes” (MG, CS, and MR), and SFB1292, individual project TP11 “Viral evasion of innate and adaptive immune cells and inbetweeners” (MR and NL).

## Conflict of Interest

The authors declare that the research was conducted in the absence of any commercial or financial relationships that could be construed as a potential conflict of interest.

## Supplementary Material

Supplementary Figure 1 Detection limits of RT-qPCRs. Limiting dilution analysis (55) based on the experimentally determined fractions of negative replicates F(0) for log_2_ graded numbers of the indicated synthetic *in vitro* transcripts (starting with 8 transcripts) subjected to RT-qPCRs in 16 replicates each. The plots of lnF(0) on the ordinate and the number of transcripts on the abscissa show the Poisson distribution regression lines calculated with the maximum likelihood method. Color-shaded areas indicate the 95% confidence intervals (CI) for the estimated most probable number (MPN). The MPN is the number of transcripts revealed as the abscissa coordinate of the point of intersection between the regression line and a line at the ordinate value lnF(0) = ln1/e = -1 (dashed lines). The null hypothesis of Poisson distribution is accepted for p >0.05.

Supplementary Figure 2 Localization of latent viral genome to EC isolated from the lungs. CD31^+^CD146^+^ EC were isolated by digestion of latently infection lung tissue followed by cytofluorometric cell sorting. Latent viral genome load was determined by qPCR specific for gene *M55*. The latent viral genome is found enriched in the sorted EC compared to all lung cells. Symbols represent triplicate measurements with the median values indicated. The analysis was performed for five latently infected mice analysed individually.

Supplementary Figure 3 Time course of MI and identification of iTEM as the “inflationary” subset of viral epitope-specific CD8^+^ T cells in the spleen. **(A)** Gating strategy for the cytofluorometric quantitation of spleen-derived CD8^+^ T cells expressing T-cell receptors specific for the pMHC-I complexes IE1-L^d^, m164-D^d^, and M105-K^d^. Control, PE-conjugated pMHC-I dextramer H-2Kb/SIINFEKL. SSC, sideward-scatter. Data refer to 6 months after HCT and infection. **(B)** Response kinetics of viral epitope-specific CD8^+^ T cells isolated from the spleen at the indicated times after HCT and infection. Shown are median values and range for five mice per time of analysis. **(C)** Response kinetics of the indicated subsets of viral epitope-specific CD8^+^ T cells isolated from the spleen at the indicated times after HCT and infection. Shown are median values and range for five mice per time of analysis. For further explanation, see the legend of **Figure 6**.
