# Supplementary figures and images for: Stochastic Episodes of Latent Cytomegalovirus Transcription Drive CD8 T-Cell “Memory Inflation” and Avoid Immune Evasion
Source: Front Immunol. 2021 Apr 22;12:668885. doi: 10.3389/fimmu.2021.668885 (PMC8100209; doi:10.3389/fimmu.2021.668885)

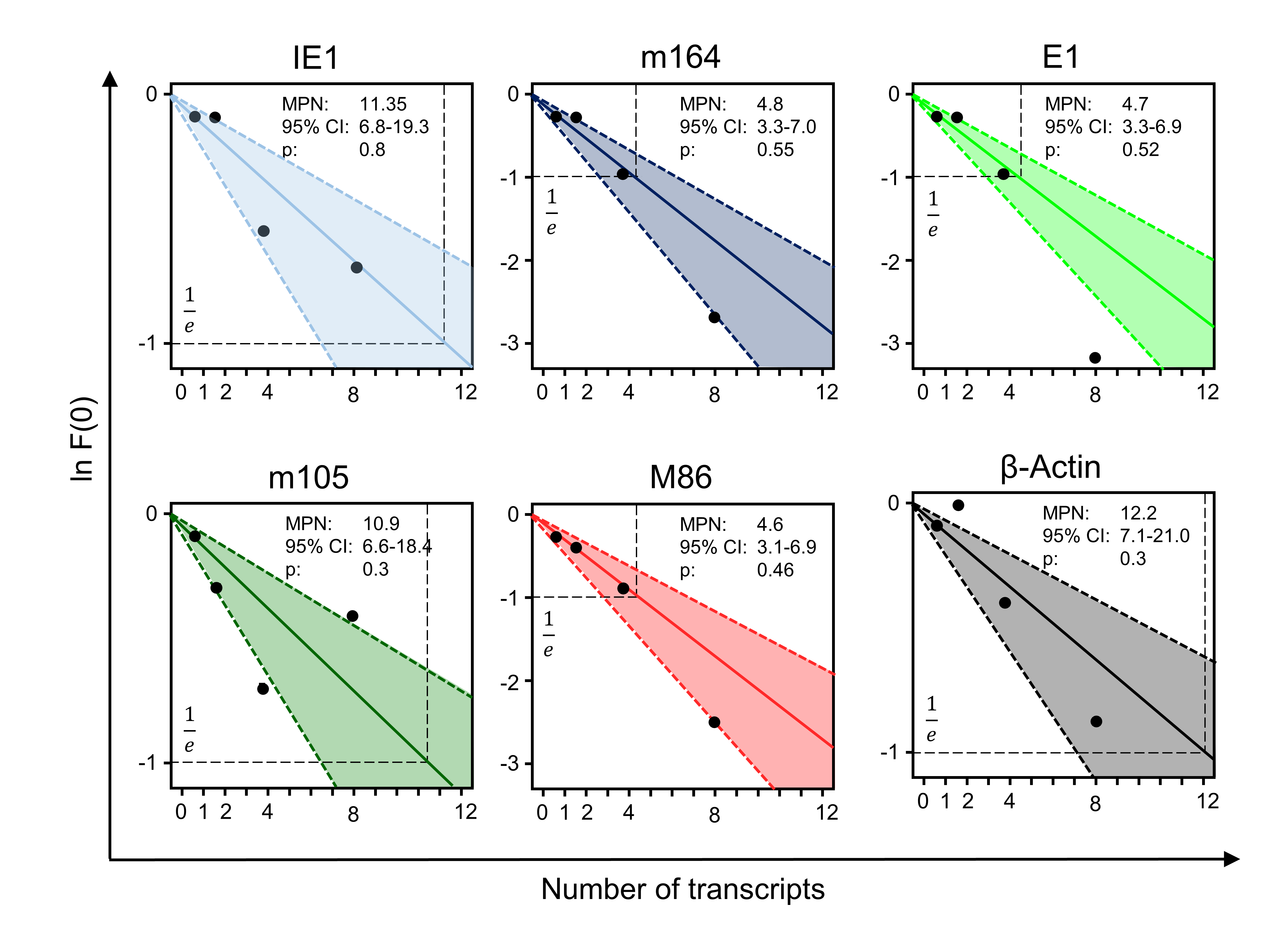

Supplement: Supplementary Figure 1 — Detection limits of RT-qPCRs. Limiting dilution analysis (55) based on the experimentally determined fractions of negative replicates F(0) for log2 graded numbers of the indicated synthetic in vitro transcripts (starting with 8 transcripts) subjected to RT-qPCRs in 16 replicates each. The plots of lnF(0) on the ordinate and the number of transcripts on the abscissa show the Poisson distribution regression lines calculated with the maximum likelihood method. Color-shaded areas indicate the 95% confidence intervals (CI) for the estimated most probable number (MPN). The MPN is the number of transcripts revealed as the abscissa coordinate of the point of intersection between the regression line and a line at the ordinate value lnF(0) = ln1/e = -1 (dashed lines). The null hypothesis of Poisson distribution is accepted for p >0.05. [file Image_1.tif]

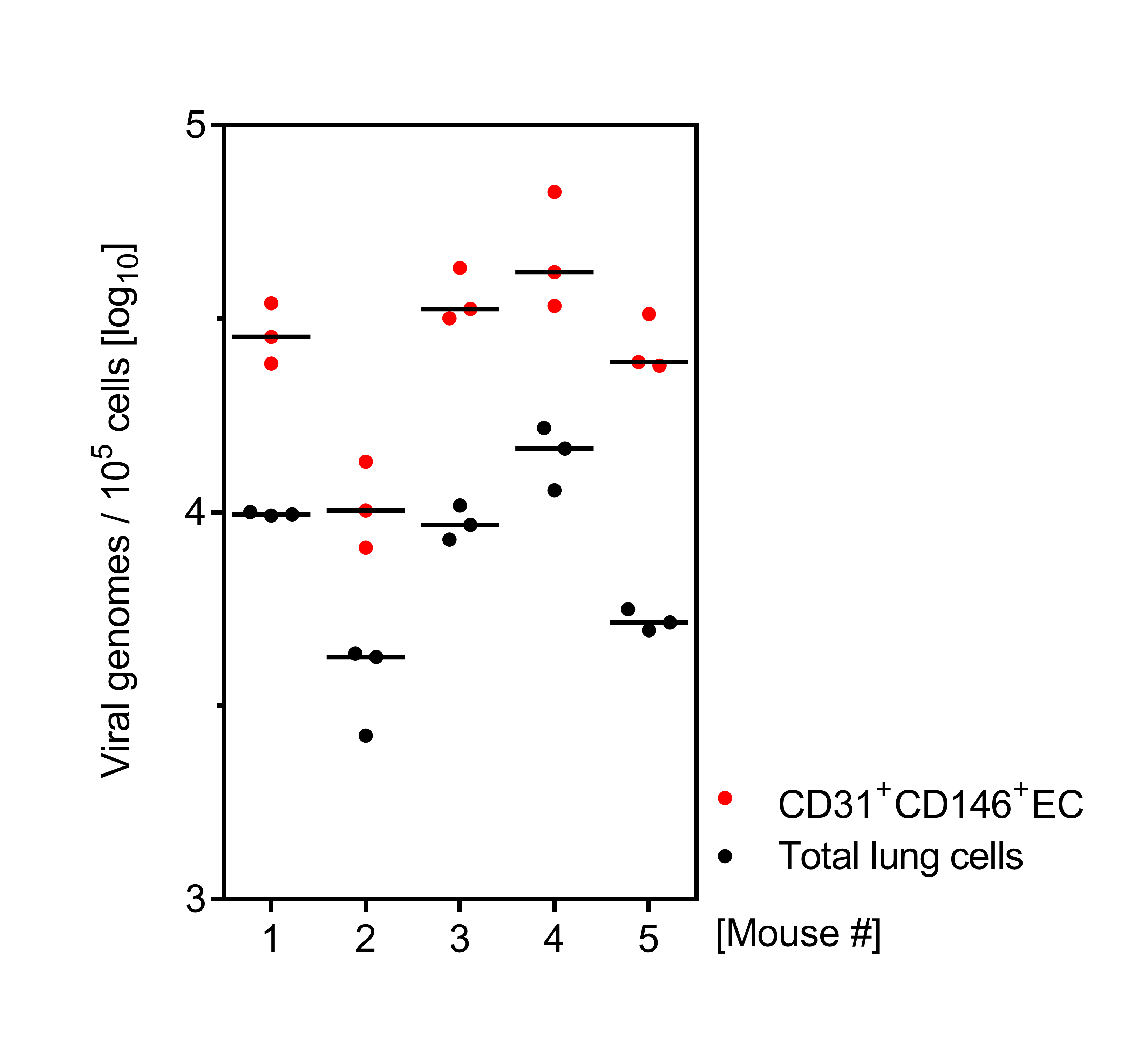

Supplement: Supplementary Figure 2 — Localization of latent viral genome to EC isolated from the lungs. CD31+CD146+ EC were isolated by digestion of latently infection lung tissue followed by cytofluorometric cell sorting. Latent viral genome load was determined by qPCR specific for gene M55. The latent viral genome is found enriched in the sorted EC compared to all lung cells. Symbols represent triplicate measurements with the median values indicated. The analysis was performed for five latently infected mice analysed individually. [file Image_2.tif]

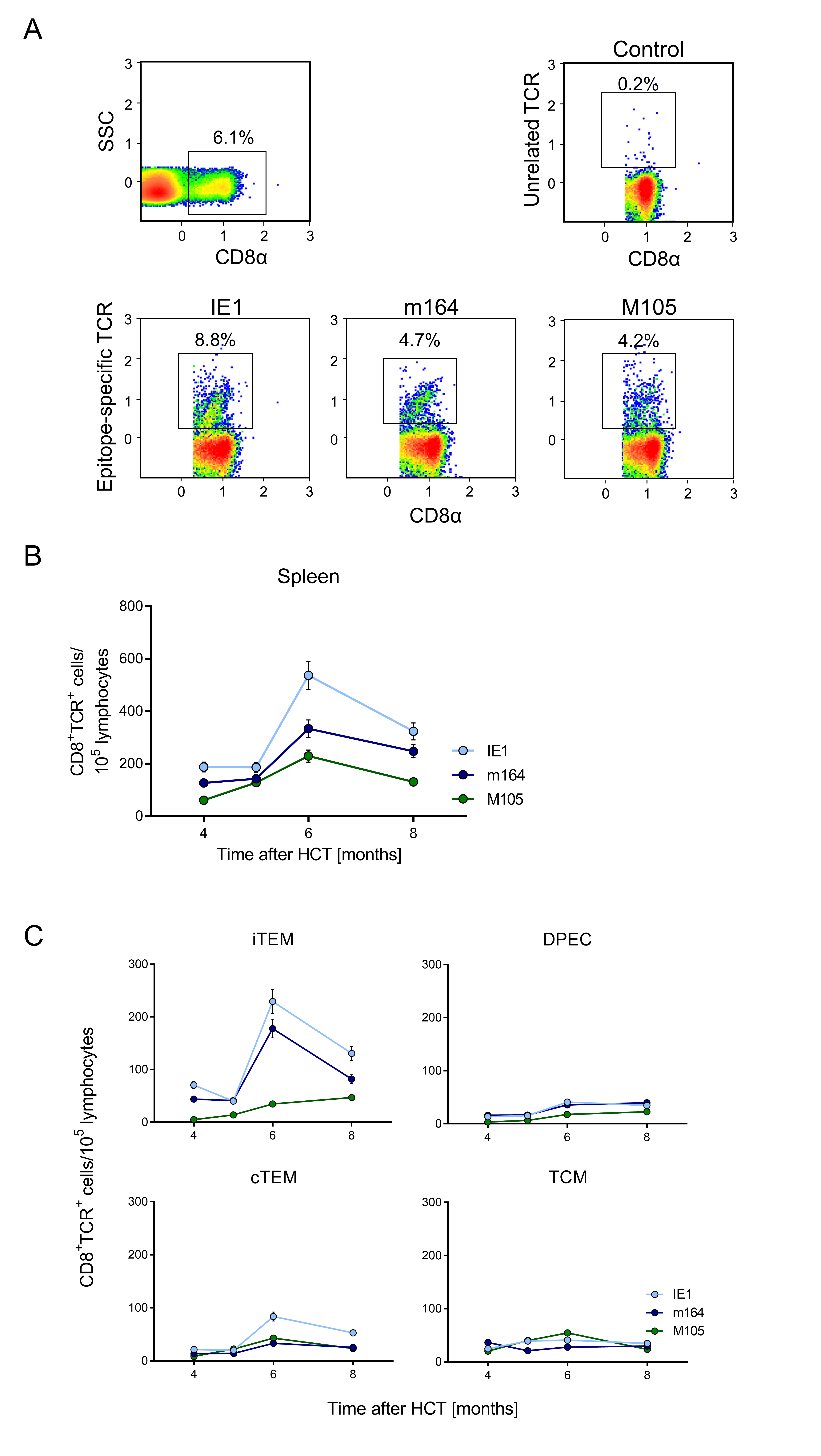

Supplement: Supplementary Figure 3 — Time course of MI and identification of iTEM as the “inflationary” subset of viral epitope-specific CD8+ T cells in the spleen. (A) Gating strategy for the cytofluorometric quantitation of spleen-derived CD8+ T cells expressing T-cell receptors specific for the pMHC-I complexes IE1-Ld, m164-Dd, and M105-Kd. Control, PE-conjugated pMHC-I dextramer H-2Kb/SIINFEKL. SSC, sideward-scatter. Data refer to 6 months after HCT and infection. (B) Response kinetics of viral epitope-specific CD8+ T cells isolated from the spleen at the indicated times after HCT and infection. Shown are median values and range for five mice per time of analysis. (C) Response kinetics of the indicated subsets of viral epitope-specific CD8+ T cells isolated from the spleen at the indicated times after HCT and infection. Shown are median values and range for five mice per time of analysis. For further explanation, see the legend of Figure 6 . [file Image_3.tif]
