# Supplementary material for: Stochastic Episodes of Latent Cytomegalovirus Transcription Drive CD8 T-Cell “Memory Inflation” and Avoid Immune Evasion
Source: Front Immunol. 2021 Apr 22;12:668885. doi: 10.3389/fimmu.2021.668885 (PMC8100209; doi:10.3389/fimmu.2021.668885)
Supplement: Supplementary file 4 [file Table_1.docx]

**Table S1 List of primers for cloning**

| **ORF** | **Name** | **Sequence** |
| --- | --- | --- |
| M112/E1 | E1_for | GACGACGTTACTTCACCTTCCG |
|  | E1_rev | GAACACATTGTCCAAGTCGACC |
| M86 | M86_HindIII_for | AAAAGCTTCGAGTAGAACCGGTGGAAGG |
|  | M86_XbaI_rev | AATCTAGAGCTCGCAGAAGGACTTACCC |
| M105 | M105_EcoRV_for | ATATGATATCGCCCTGGTTCGTCACGTTCC |
|  | M105_SphI_rev | ATATGCATGCCAGACCGATGCCCTGACTCTTG |
| m152 | m152_HpaI_for | GGAGTTAACCATATAAAAGCTGTCCCCCATGCCATTCG ATCAGACGCGGGCTACTCCCGAAAGAGTAAC |
|  | m152-HpaI_rev | GGAGTTAACTGACTAATAAGTTATCTTTATTGTACAAGTGT TGTGTGTTATCCCTGAGCCCATTCCCAG |
