# Supplementary material for: Stochastic Episodes of Latent Cytomegalovirus Transcription Drive CD8 T-Cell “Memory Inflation” and Avoid Immune Evasion
Source: Front Immunol. 2021 Apr 22;12:668885. doi: 10.3389/fimmu.2021.668885 (PMC8100209; doi:10.3389/fimmu.2021.668885)
Supplement: Supplementary file 5 [file Table_2.docx]

**Table S2 List of primers and probes for qPCR and RT-qPCR**

| **Target** | **Name** | **Sequence** |
| --- | --- | --- |
| E1/M112 | IE1_for | TGGCTGATTGATAGTTCTGTTTTATCA |
|  | IE1_rev | CTCATGGACCGCATCGCT |
|  | IE1_probe | AACGCTCCTCACTGCAGCATGCTTG |
| IE1/m123 | E1_for | TCGAAGAGGAATGTTCTCCACG |
|  | E1_rev | TTGTTGTCCTCCATCGCTGA |
|  | E1_probe | AGCCCAAGCGCCAGAAGACCCA |
| M86 | M86_for | GGTCGTGGGCAGCTGGTT |
|  | M86_rev | CCTACAGCACGGCGGAGAA |
|  | M86_probe | TCGGCCGTGTCCACCAGTTTGATCT |
| M105 | M105_for | CCAGGTATCGGATCATGCAGAAG |
|  | M105_rev | TGGGACTCGATCTCGCAGTTC |
|  | M105_probe | ACCCGGCTCGTCACCTACAACAGGA |
| m152 | m152_for | CGTTCGCGAGACTGATGTTGT |
|  | m152_rev | GCAACGGCTACGTGTCCTGTA |
|  | m152_probe | CCAACGGAACCTGAGTGCGCA |
| m164 | m164_for | CAACTGACAGTCGCAGCTCTTC |
|  | m164_rev | CGGCGGTAACCTGCTATCC |
|  | m164_probe | TCGGCCGTGTCCACCAGTTTGATCT |
| β-actin | β-actin_for | GACGGCCAGGTCATCACTATTG |
|  | β-actin_rev | CACAGGATTCCATACCCAAGAAGG |
|  | β-actin_probe | AACGAGCGGTTCCGATGCCC |
| M55 | M55_for | GGTATCTGCCCTCATCGTCTG |
|  | M55_rev | AATCCGTCCAACATCTTGTCG |
| PTHrP | PTHrP_for | GGTATCTGCCCTCATCGTCTG |
|  | PTHrP_rev | GGTATCTGCCCTCATCGTCTG |

All probes were labeled [5′ 6-FAM labeled, 3′ black hole quencher (BHQ) labeled]
